# Distribution, Burden, and Impact of Acute Gastroenteritis in Dominica, 2009-2010

**Published:** 2013-12

**Authors:** Shalauddin Ahmed, Paul Ricketts, Marc Bergeron, Walter Jones, Lisa Indar

**Affiliations:** ^1^Health Information Unit, Ministry of Health, Commonwealth of Dominica; ^2^Ross University School of Medicine, Dominica; ^3^Princess Margaret Hospital Laboratory, Dominica; ^4^Caribbean Epidemiology Centre, 16-18 Jamaica Boulevard, Federation Park, Port of Spain, Trinidad and Tobago

**Keywords:** Acute gastroenteritis, Disease burden, Prevalence, Dominica

## Abstract

Acute gastroenteritis (AGE) is an important public-health issue in Dominica. To determine the burden of AGE in Dominica, a retrospective, cross-sectional population survey was conducted in March-April 2009 and October 2010 (low- and-high-AGE seasons) and a laboratory survey from April 2009 to March 2010. The overall monthly prevalence of self-reported AGE was 8.6 % (95% CI 7.0-10.6); the incidence rate was 1.1 episodes/person-year and 79,157.1 episodes of AGE for the total population/year. Monthly prevalence of AGE was the highest in the 1-4 year(s) age-group (25.0%), higher in females (10.8%) and also varied by health district, with the highest monthly prevalence of AGE being reported in the Portsmouth district (13.1%). This difference in gender and across the health region was statistically significant. The estimated underreporting of syndromic AGE to the Ministry of Health was 83.3%. Furthermore, for every reported laboratory-confirmed case of AGE and foodborne disease (FBD), there was an estimated underreporting factor of 280. Overall, 47% of AGE specimens tested were positive for FBD pathogens. The predominant pathogens isolated were norovirus, followed by *Giardia, Salmonella,* and *Shigella.* The total annual estimated cost of AGE was US$ 1,371,852.92, and the total cost per capita due to AGE was US$ 19.06, indicating an economic burden of AGE-related illness on a small island of Dominica.

## INTRODUCTION

The Commonwealth of Dominica, situated between the two French Islands of Martinique and Guadeloupe, has a land area of 298 square miles and a total population of 71,293 (1). It is divided into 10 parishes and 7 health districts, with the most densely-populated parish being St. Georges; St. David parish is mostly rural. Primary healthcare services are delivered through facilities throughout the island via its seven health districts, each with a peripheral network of clinics and health centres/clinics. The Health Information Unit (HIU) is the disease surveillance unit for the Ministry of Health (2).

Acute gastroenteritis (AGE) is defined as the passage of three or more loose or liquid stools per day and isalso known as acute diarrhoeal disease. It has long been recognized as a leading cause of child morbidity and mortality in the world; about two billion cases of diarrhoeal diseases are reported globally every year (3). The recent outbreak of cholera in Haiti reinforced acknowledgement of the ongoing threat of AGE in the Caribbean (4). Data collected by the Caribbean Epidemiology Centre (CAREC) from its member countries show high and increasing numbers of reported cases of AGE (5); however, the exact distribution and burden of AGE is not fully known at this time.

In Dominica, there are syndromic and laboratory-based surveillance systems for AGE. These data are reported weekly to Health Information Unit of the Ministry of Health. Syndromic AGE data in Dominica indicate that AGE is the second-most common communicable disease syndrome after acute respiratory illness (ARI) and that 1,510 and 1,020 cases of AGE (in <5 years and >5 years age-groups) were reported in 2006 and 2007 respectively (6). Data are collected from all government health centres and selected sentinel sites. Over the last five years, Dominica has had three outbreaks of AGE. Norovirus was identified in the 2006 and 2010 AGE outbreaks. In 2008, rotavirus was identified as the aetiologic pathogen (6).

It is well-known that reported AGE represents only a small fraction of the total AGE in the community. Thus, to obtain a comprehensive picture of the distribution and burden of AGE and foodborne diseases in Dominica, the Ministry of Health, in collaboration with Caribbean Epidemiology Centre (CAREC), Pan American Health Organization (PAHO), and Ross University (RUSM), conducted a burden of illness study in Dominica during 2009-2010. Additional technical and financial assistance was also provided by the Public Health Agency of Canada (PHAC) and the Caribbean Eco-Health Programme (CEHP). This study was part of the Caribbean Burden of Illness Study being conducted in seven other countries. The result of this study will be used in advocating for resources to improve AGE and FBD surveillance and in designing the appropriate intervention measures.

## MATERIALS AND METHODS

### Population survey

A retrospective, cross-sectional population survey was conducted in 2 phases in all 7 health districts, using the household list maintained by the Central Statistical Office as the sampling frame. The first phase was conducted in 22 March–4 April 2009 (low-AGE season) and 17–31 October 2010 (high-AGE season). The high- and low-AGE seasons were designated based on the average 5-year syndromic AGE surveillance data. In the selected households, the individuals were interviewed on the day before the next birthday. Residents of Dominica, aged higher than 1 year, were included while persons of less than 1 year of age, those not living in Dominica at the time of the survey, prisoners, mentally-disabled persons, and those who did not consent or were unwilling to participate were excluded.

### Sample-size

A sample-size of 1,204 was calculated using Epi Info (version 3.5.3) based on a population of 71,008, an expected prevalence of AGE of 15%, allowable error of 2%, and 95% confidence interval. This number was rounded to 1,210; and so, 605 interviews were conducted in each study period.

### Data collection, validation, and security

The survey questionnaire was administered during face-to-face interviews by trained student nurses and community health nurses in Phase I and Phase II respectively. Verification of the interviews was done by telephone calls to 10 randomly-selected households in each phase. Respondents were asked if they had experienced any symptoms of diarrhoea in the past 4 weeks, with the case definition of diarrhoea being three episodes of loose stools (taking the shape of the container) in 24 hours. Additional questions were asked about sociodemographic factors, behavioural factors, secondary symptoms, whether cases sought medical care in public or private facilities, the use and type of medications, number of missed school and/or work days, and whether hospitalization was required.

### Data-entry and analysis

Data were double-entered into a database developed in EpiData (version 3.1), and the two datasets were compared for validation. Analysis was performed using Epi Info (version 3.5.1) software (Centers for Disease Control and Prevention, USA) as well as Microsoft Excel 2007 (Microsoft Corporation, One Microsoft Way, Redmond, WA 98052-6399, USA). Individuals responding ‘do not know’ or those who gave no response to any question were excluded from the analysis of that question. Univariate analysis was performed on the overall dataset. The null hypothesis of no association between the presence of AGE and the sociodemographic factors was tested using the chi-square test for statistical significance. The burden and underreporting factors for AGE were estimated using syndromic and laboratory-confirmed AGE data reported to the Ministry of Health and collected from the population and laboratory surveys.

### Laboratory survey

The laboratory survey was conducted from April 2009 to March 2010 at the Princess Margaret Hospital Laboratory, the only government medical laboratory on the island and processed approximately 90% of AGE stool specimens for the island. All submitted diarrhoeal stool samples were tested for *Salmonella, Shigella, Campylobacter, E. coli* (if the sample was bloody), norovirus, *Giardia, Cryptosporidium,* and *Entamoeba histolytica* parasites, using standard methods. During a part of the study period, from 17 January to 27 February of 2010, an outbreak of norovirus was detected.

### Ethical approval

The study was approved by the National Human Research Ethics Committee of Dominica. An oath of confidentiality was also obtained from each interviewer to conduct the population survey. All data collected were kept confidential. The participants were identified by code and not by name on the questionnaire. Participants were informed about the purpose of the survey and asked to sign a consent form before the questionnaire was administered.

### Estimation of underreporting and burden of syndromic and laboratory-confirmed AGE

Data on syndromic and laboratory-confirmed AGE reported to the Health Information Unit of the national surveillance were compared with data from the population and laboratory surveys (including the reporting and underreporting levels that occur in the country from the time when a person is ill with AGE/diarrhoeal disease to the time when it is documented in the national surveillance system). Data from the surveys include information on seeking medical care, request for stool specimen, submission of stool specimen, and testing of stools. These are used in producing national estimates of the burden and extent of underreporting for syndromic AGE and laboratory-confirmed FBD/AGE pathogens, using the models shown in the burden of illness pyramid.

### Estimation of economic and social impact

The economic burden of AGE was estimated using data from the population survey, e.g. percentage seeking medical care in public and private healthcare system, taking medication, type of meditation, hospitalization, adults losing median number of work days, and adults requiring caregiver for median number of days during his/her illness. The cost of medical supplies in public healthcare was obtained from the Central Medical Store whereas an average cost was used for private services and supplies from private clinics and dispensaries (7).

## RESULTS

### Response rate and representativeness of respondents

Of the total residents in 1,210 randomly-selected households, 973 individuals were contacted—478 in Phase 1 and 495 in Phase 2—with an overall response rate of 80.4%. Comparison of the demographic profile of Dominican residents (general population) and the survey respondents indicates that, overall, respondents were older than the census population and were more likely to be female (Table 1).

### Magnitude of illness

Of the 973 respondents, 91 (9.4%) reported that they had sudden onset of diarrhoea (3 or more watery or loose stools within 24 hours with or without fever, vomiting, or visible blood in the stool) in the 4 weeks prior to the interview and were, therefore, classified as self-reported cases of acute gastroenteritis (AGE). Of these 91 cases, 7 (7.7%) stated their symptoms were due to pre-existing/chronic conditions. Since the objective of the study was to describe AGE illnesses, these 7 respondents were included in the non-case group. Of the 84 remaining cases of AGE, 20 (23.8%) reported more than one episode in the 28 days prior to the interview. The period prevalence of self-reported AGE was 8.6% (95% CI 7.0-10.6). The yearly incidence rate was 1.1 episodes per person-year (Table 2). The monthly prevalence for the high- and low-AGE seasons observed previously was not significantly different (low-AGE season 9.2%, 95% CI 6.8–12.2; high-AGE season 8.0%, 95% CI 5.9-10.9).

### Distribution of illness

The monthly prevalence of AGE by age-group, gender, education of male and female household heads, ethnic group, and monthly household income are outlined in Table 1. Using univariate analysis, the monthly prevalence of AGE was found higher among females (10.8%) than males (6.7%). This difference was statistically significant (p=0.04).

**Figure 1.**
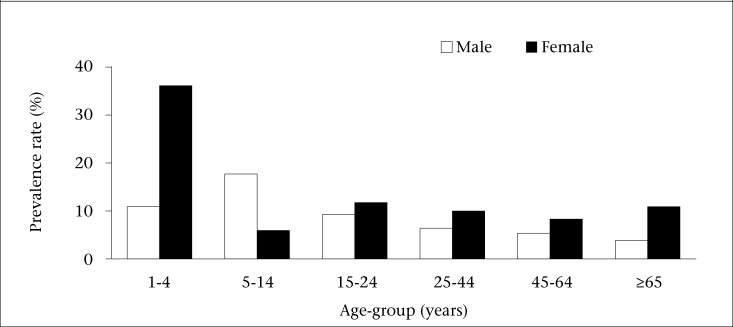
Monthly prevalence of AGE by age-group and sex in Dominica

The highest monthly prevalence of AGE was among the 1-4 year(s) age-group (25.0%), followed by 15-24 years (10.6%), 5-14 years (10.4%), and over 65 years age-group (9.5%) (Figure 1). These differences were not statistically significant (p=0.14). The age- and gender-adjusted monthly prevalence of AGE were 9.9% and 8.4% respectively. There was no significant difference between the crude and age-adjusted monthly prevalence of AGE and gender-adjusted monthly prevalence of AGE.

**Figure 2.**
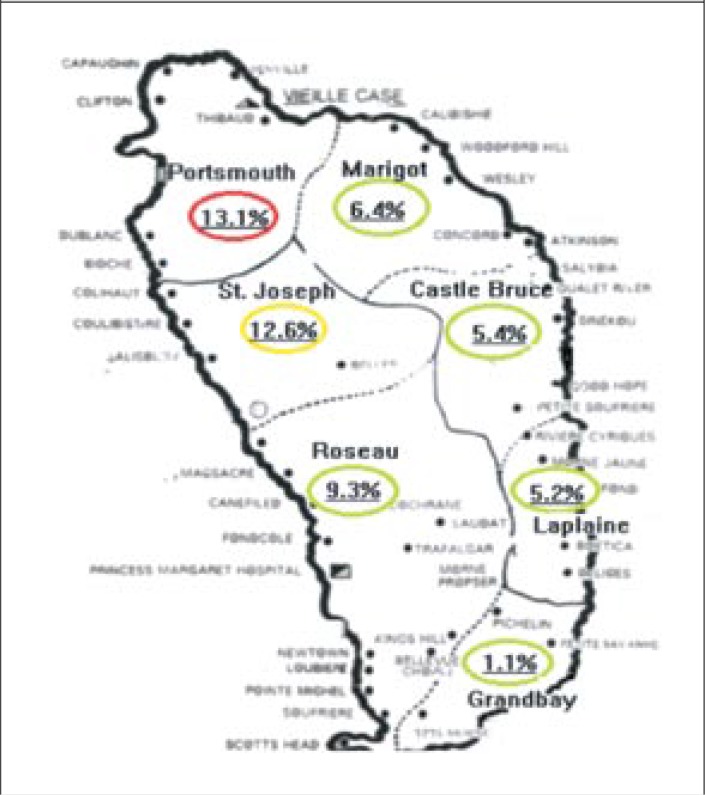
Monthly prevalence of AGE in health districts

Prevalence of AGE also varied by health district, with the highest monthly prevalence of self-reported cases of AGE being reported in the Portsmouth district (13.1%), followed by St. Joseph (12.6%) and Roseau district (9.3%) and the lowest reported in Grand Bay (2.1%) (Figure 2). This difference across the health regions was statistically significant (p=0.01).

**Table 1.** Demographic characteristics of general population and survey respondents and monthly prevalence of self-reported acute gastroenteritis per category in Dominica, 2009-2010

Variable	Residents (N=71,961)	Respondents (n=972) No. (%)	Monthly prevalence of AGE% (No.)	95% Confidence interval
Sex (p=0.04)				
Male	36,551	431 (44.3)	6.7 (29)	4.6-9.6
Female	35,410	541 (55.7)	10.8 (55)	7.8-13.1
Age (completed years) (p=0.14) (n=966)				
1-4	5,167	20 (2.1)	25 (5)	8.7-49.1
5-14	14,798	77 (8.0)	10.4 (8)	4.6-19.4
15-24	11,504	94 (9.7)	10.6 (10)	5.2-18.7
25-44	20,751	281 (29.1)	8.2 (23)	5.3-12.0
45-64	11,131	284 (29.4)	7.0 (20)	4.4-10.7
≥65	7,346	210 (21.7)	8.1 (17)	4.8-12.6
Ethnic group (p=0.71) (n=946)				
African/Black	Not available	874 (92.4)	8.6 (75)	6.8-10.7
Indian	Not available	3 (0.3)	0.0	0.0-70.8
Asian	Not available	3 (0.3)	0.0	0.0-70.8
European	Not available	3 (0.3)	33.3 (1)	0.8-90.6
North American	Not available	10 (1.1)	10.0 (1)	0.3-44.5
Other (Indigenous)	Not available	53 (5.6)	7.5 (4)	2.1-18.2
Monthly income (EC$) (p=0.37) (n=807)				
Low income (0-1,000)	Not available	456 (56.6)	9.0 (41)	6.6-12.1
Medium income (1,001-2,000)	Not available	186 (23.0)	6.5 (12)	3.4-11.0
High income (>2,000)	Not available	165 (20.4)	10.9 (18)	6.6-16.7
Education (Mother) (p=0.12) (n=796)				
Primary	Not available	501 (62.9)	9.4 (47)	7.0-12.4
Secondary	Not available	156 (19.6)	8.3 (13)	4.5-13.8
Certificate/Diploma	Not available	88 (11.1)	8.0 (7)	3.3-15.7
Graduate/Undergraduate	Not available	29 (3.6)	24.1 (7)	10.3-43.5
Postgraduate	Not available	16 (2.0)	12.5 (2)	1.6-38.3
Education (Father) (p=0.11) (n=658)				
Primary	Not available	426 (64.7)	8.2 (35)	5.9-11.3
Secondary	Not available	128 (19.5)	3.9 (5)	1.3-8.9
Certificate/Diploma	Not available	62 (9.4)	6.5 (4)	1.8-15.7
Graduate/Undergraduate	Not available	15 (2.3)	20.0 (3)	4.3-48.1
Postgraduate	Not available	21 (3.2)	0 (0)	0.0-16.1
Health districts (p=0.0144)				
Portsmouth	8,515	160	13.1 (21)	8.3-19.4
St. Joseph	6,129	95	12.6 (12)	6.7-21.0
Roseau	36,017	388	9.3 (36)	6.7-12.7
Marigot	8,335	125	6.4 (8)	2.8-12.2
Castle Bruce	3,898	127	5.4 (3)	1.1-14.9
La Plaine	3,389	58	5.2 (3)	1.1-14.4
Grandbay	5,678	94	1.1 (1)	0.0-5.8

### Symptoms and severity

In the 84 cases, the most common secondary symptoms included nausea (28.6%), followed by headache (25.0%), cough (25.0%), runny nose (20.2%), sneezing (17.9%), and vomiting (14.3%) (Table 3). The maximum number of stools per 24 hours ranged from 3 to 9, with a median of 4. The average number of days an individual suffered from AGE was 2.5, with a range of 1-28 day(s) and a median of 2 days (Table 2). Of the 84 cases, 42 reported restricted activity and had to spend time at home due to their illness. The range of days spent at home was 1-7, with a median of 1.5 days (Table 3). Fourteen cases required other individuals to look after them while ill. The range of days taking care of a case was 1-28, with a median of 2 days.

**Table 2.** Minimum set of results proposed for studies of AGE, Dominica, 2009-2010

Category	Cases
Annual incidence/person-year	1.1
Annual incidence/person-year in males	1.0
Annual incidence/person-year in females	1.4
Cases with bloody diarrhoea (%)	1 (1.3%)
Cases that saw a physician (%)	14 (16.7%)
Cases who submitted a sample for testing (%)	2 (40%)

### Healthcare-seeking behaviours

Of the 84 cases, 14 (16.7%) sought medical care for their illness. Six attended an outpatient clinic in a public hospital, 6 attended a health centre, and 2 went to a private physician. No cases reported having been hospitalized. Five cases had a stool specimen requested for (Table 4). Eight out of 14 cases who sought medical care reported having medication prescribed. Three were prescribed antibiotics, two of whom completed the course of antibiotics and one of whom did not, for an unknown reason. Four individuals were prescribed oral rehydration solution. Three were prescribed vitamins and antihelminthic medication. Thirty-seven cases took non-prescribed medications for their illnesses. Of them, 24 took ‘unknown bush medicine’.

### Risk factors, habits, and hygiene

Individuals were asked to identify what they believed to have caused their illness. Twenty-five (29.8%) cases believed they became ill from food consumption; 19 (22.6%) believed that water was the cause; and 5 (6.0%) believed contact with another sick person caused the disease. The number of individuals living in the households of respondents ranged from 1 to 13, with a median of 3. Households of non-cases and cases had a median of 3. This difference was not significant. Additionally, 19 cases reported that another individual was ill with diarrhoea in their home at the same time of their illness. Three reported 2 additional individuals to be ill, and four reported 3 others to be ill. Less than 10% of respondents reported washing their hands with or without soap before meals and after going to the toilet. However, handwashing was not significantly associated with being a case of AGE (Table 5). Out of 973 respondents, 9 went for swimming in the ocean, 7 went for swimming in the river, and 4 for swimming in the pool in the month prior to the interview. Swimming in the ocean, river or pool was not significantly associated with AGE (p=0.86, 0.98, 0.06 respectively). Of the 84 cases who purchased uncooked chicken, 72 cases purchased their chicken frozen, 6 purchased chilled, 5 did not purchase chicken, and 1 purchased live chicken (Table 6). Impact of purchased chicken was statistically insignificant (p=0.30).

**Table 3.** Secondary symptoms, duration, and severity of symptoms in respondents in Dominica, 2009-2010

Secondary symptom (n=84)	No. of cases	%	95% CI	
Nausea	24	28.6	19.2-39.5	
Headache	21	25.0	16.2-35.6	
Vomiting	12	14.3	7.6-23.6	
Abdominal pain	2	2.4	0.3-8.3	
Blood in stool	1	1.2	0.0-6.5	
Fever (measured)	5	6.0	2.0-13.3	
Fever (not measured)	9	10.7	5.0-19.4	
Sore throat	6	7.1	2.7-14.9	
Cough	21	25.0	16.2-35.6	
Runny nose	17	20.2	12.3-30.4	
Sneezing	15	17.9	10.4-27.7	
Duration		Mean (day)	Median (day)	Range (day)
Duration of illness		2.5	9	1-28
Activity restricted to home		2.3	1.5	1-7

**Table 4.** Healthcare-seeking behaviours in relation to AGE

Category	Number	%	95% CI
Number of cases seeking medical care (n=84)	14	16.7	9.4-26.4
Number of cases asked to submit specimen (n=14)	5	35.7	12.8-64.9
Number submitting specimen (n=5)	2	40.0	5.3-85.3
Number of cases prescribed medications (n=14)	8	57.1	28.9-82.3
Number taking antibiotics (n=14)	3	21.4	4.7-50.8
Number taking oral rehydration fluid (n=14)	4	28.6	8.4-58.1
Number taking non-prescribed medications (n=84)	37	44.0	33.2-55.3
Number of stools in 24 hours	4.16 (Mean)	4 (Median)	3-9 (Range)

**Table 5.** Handwashing practices

Handwashing pattern	No. of survey respondents	No. of cases	%	95% CI
Before meals (with or without soap)	549	48	8.7	6.6-11.5
Before meals (with soap)	535	48	9.0	6.8-11.8
After going to the toilet (with or without soap)	804	70	8.7	6.9-10.9
After going to the toilet (with soap)	704	58	8.2	6.4-10.6

### Laboratory survey

During April 2009 to March 2010, 73 diarrhoeal/AGE stool samples were tested, and 47% were positive for an FBD pathogen—29% were positive for norovirus, 7% for *Salmonella,* 12% for *Giardia,* and 4% for *Shigella.* The laboratory practices and data are outlined in Table 7 and 8.

### Estimating the underreporting and burden of AGE in Dominica

**Figure 3.**
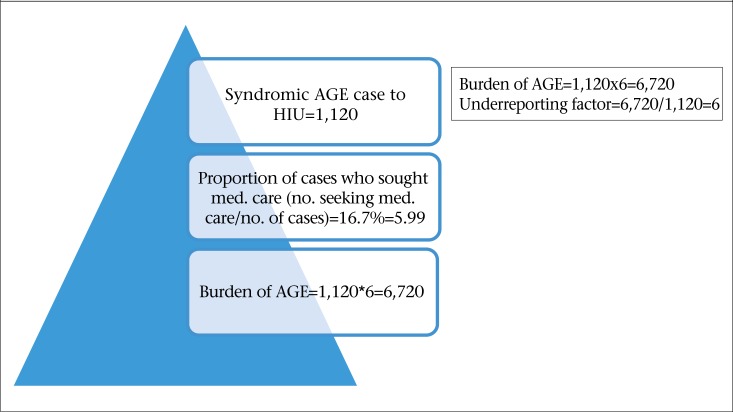
Estimating burden of AGE for the period April 2009–March 2010, using syndromic surveillance data

**Figure 4.**
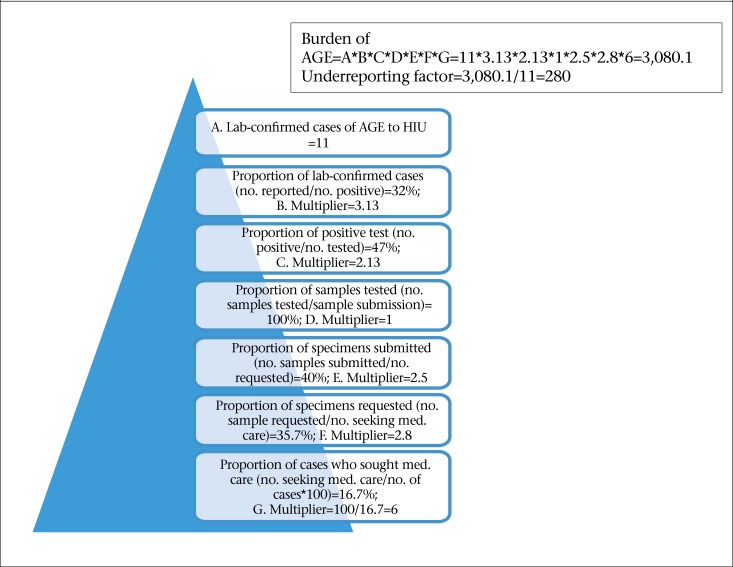
Estimating burden of laboratory–confirmed AGE for the period April 2009–March 2010 using lab–confirmed cases

The distribution of the proportions of cases reported at each step in the reporting chain for AGE is outlined in Table 9. This input distribution was determined using syndromic and laboratory-confirmed AGE data reported to the HIU, Ministry of Health and data from the population and laboratory surveys. Based on syndromic AGE data, the estimated burden of AGE for one year period (April 2009–March 2010) in Dominica was 6,720. The number of syndromic AGE cases reported to the Ministry of Health for the specified period was 1,120. Thus, there was an underreporting factor of 6 (6,720/1,120), (Figure 3). The overall underreporting rate for AGE in Dominica is 6 for every 1 reported case of AGE to the Ministry of Health. Using laboratory surveillance data, the estimated burden of laboratory-confirmed AGE for the one year period (April 2009–March 2010) in Dominica was found to be 3,080. The number of cases reported to Health Information Unit for the specified period was 11. There is an underreporting factor of 280 (3,080/11) for laboratory-confirmed foodborne/AGE pathogens in Dominica (Figure 4). The overall underreporting rate for laboratory-confirmed FBD/AGE pathogens in Dominica is 280 for every 1 case reported to the Ministry of Health.

**Table 6.** Respondents’ exposures to high-risk food items in the burden of AGE in Dominica

Purchased uncooked chicken	Respondents	No. of cases (n=84)	Overall monthly prevalence (%)	95% CI
Live	48	1	2.1	0.1-11.1
Frozen	746	72	9.7	7.7-12.1
Chilled	79	6	7.6	2.8-15.8
Did not purchase	79	5	6.3	2.1-14.2

**Table 7.** Laboratory testing data, burden of AGE, Dominica (April 2009–December 2010)

Month	Total number of diarrhoeal samples submitted	Total number of diarrhoeal samples tested	Total number of samples testing positive	Number reported	Positive other non-Typhi *Salmonella*	Positive for *Shigella sonnei*	Positive for norovirus	Positive for parasites
Apr-09	15	15	6	0	-	-	2	4
May-09	8	8	3	1	1	-	2	
Jun-09	12	12	8	4	2	2	5	1
Jul-09	6	6	1	-	-	-	-	1
Aug-09	6	6	5	5	1	1	2	3
Sep-09	6	6	0	-	-	-	-	-
Oct-09	2	2	0	-	-	-	-	-
Nov-09	2	2	0	-	-	-	-	-
Dec-09	0	0	0	-	-	-	-	-
Jan-10	5	5	4	-	-	-	4	-
Feb-10	8	8	6	-	-	-	6	-
Mar-10	3	3	1	1	1	-	-	-
Total	73	73	34	11	5	3	21	9
Percentage	-	100	47	32	7	4	29	12

**Table 8.** Laboratory practices by pathogen

Laboratory test done on diarrhoeal stool	Sensitivity of test (%)	How often does lab test done for pathogens (%)	Actual lab data collected	Number reported to surveillance unit of MOH	Proportion (%) of pathogens reported to the surveillance unit, MOH
*Salmonella*‡	95	95	5	5	100
*Shigella*‡	95	95	3	3	100
Campylobacter*	95	45	0	-	-
*E. coli* 0157:H7	95	20	0	-	-
*S. aureus*	95	0	0	-	-
*Vibrio*	95	0	0	-	-
Rotavirus†	95	55	0	-	-
Norovirus†	95	55	21	1	4.8
Parasites‡	75	95	9	2	22.2

*Testing for *Campylobacter* was done intermittently from September 2009 to December 2010;

†Norovirus/Rotavirus kits were not always available (Samples forwarded to CAREC);

‡Laboratory testing for *Salmonella/Shigella* remained predominant; however, recovery appeared to improve after enhanced laboratory methods; Parasites include *Giardia, Cryptosporidium,* and *Entamoeba histolytica*

**Table 9.** Information for calculation of burden of AGE and specific pathogens

Information	Source and contact person	Value
No. of syndromic AGE cases reported to MOH (Apr 2009–Mar 2010)	Ministry of Health Surveillance Unit	1,120
Proportion of laboratory-confirmed cases^1^ reported to MOH (Apr 2009–March 2010)	Ministry of Health Surveillance Unit	32%
Number of laboratory-confirmed cases isolated at the laboratory (Apr 2009–March 2010)	Local laboratory director	11
Proportion of positive cases of AGE^2^	Local laboratory director	47%
Proportion of AGE samples tested at laboratory	Laboratory	100%
Proportion of AGE samples submitted to the laboratory	Population survey	40%
Proportion of AGE cases seeking medical care	Population survey	16.7%
Number of AGE cases in population survey (meeting AGE case definition)	Population survey	84

^1^Includes all AGE-related pathogens (bacterial, viral, parasitic);

^2^AGE specimen: diarrhoeal/AGE stool specimens only (exclude hard stool specimens)

### Estimating economic burden of AGE

There are direct and indirect costs as a consequence of AGE. We were only able to estimate the direct cost due to the limited data available for calculating indirect cost in public health system (e.g. daily services of doctors, nurses, and others). The economic burden of AGE was estimated using cost data from both public and private healthcare systems. The total annual estimated cost of AGE was EC$ 3,727,187 (US$ 1,371,852.92) (Table 10-12). The expense through the public healthcare system was EC$ 3,582,766 (US$ 1,318,696.31) and through the private healthcare system was EC$ 144,421 (US$ 53,156.54) (Table 12). The total cost per capita due to AGE is EC$ 51.8/year (US$ 19.06). Most of the cost (EC$ 2,793,766/US$ 1,028,291.8 at minimum wage) was incurred due to loss of working days as a result of AGE (Table 12) .

**Table 10.** Minimum cost–related information for calculation of economic burden of AGE

Multiplier factor	Source	Value
Percentage of cases seeking medical care in private healthcare system	Population study	2/84×100=2.4%; these two also sought medical care in public health system
Percentage of cases seeking medical care in public hospitals	Population study	6/84×100=7.1%
Percentage of cases taking pain killers in public/private system*	Population study	4/84×100=4.8%
Percentage of cases taking ORS in public/private system*	Population study	4/84×100=4.8%
Percentage of cases taking antibiotics in public/private system*	Population study	3/84×100=3.6%
Percentage of cases taking Gravol (antiemetic) in public/private system*	Population study	1/84×100=1.2%
Percentage of cases taking Loperamide (antidiarrhoeal) in public/private system*	Population study	1/84×100=1.2%
Percentage of cases taking IV fluid in public/private system*	Population study	0**
Percentage of cases submitting stool specimens*	Population study	2/84×100=2.4%
Loss of working days		
Median no. of days***	Population study	1.5 days
Percentage of adults staying home during illness	Population study	62.7%****
Caregiver service		
Median no. of days***	Population study	2 days
Percentage of cases needing a caregiver	Population study	14/84×100=16.7%
Number of cases who had acute gastroenteritis		84

*There was no data to differentiate between the percentages of those who purchased their medical supplies in public healthcare system and those in private healthcare system. Taking into consideration that those who sought medical care in private clinics (2.2%) also sought care from public health system, the cost of medical supplies was calculated using cost from the public health system only;

**There were no cases of hospitalization; hence, it is unlikely to have had IV fluid, and hospitalization cost is not included in the estimation;

***As the distribution of loss of working days was skewed, median value was used rather than mean;

****20-64 years age-group was considered ‘working group’. There was no data on occupation

## DISCUSSION

The purpose of this study was to estimate the burden and demographic distribution of acute gastroenteritis (AGE) in Dominica. It was the first study of its kind in Dominica. The high underreporting of syndromic AGE (83%) and laboratory-confirmed AGE (99.6%), high national economic cost of EC$ 3,727,187.19 (US$ 1,371,852.92), and high cost per capita of EC$ 51.8/year (US$ 19.06) due to AGE have provided very strong evidence to indicate that AGE and foodborne diseases (FBD) pose a health, economic and social burden on the population of Dominica. Our result demonstrated that the true burden of AGE is much larger than is detected by the current syndromic and laboratory-based surveillance systems for AGE, implying gaps in these systems. Many more cases of AGE are occurring in the population of Dominica than are reported, and much more money is being spent on AGE-related illness than is being reported. These results should, therefore, be used in advocating for resources to improve AGE and to design appropriate interventions to reduce the burden of AGE in Dominica.

The prevalence of AGE in our study (8.6%) was lower than the prevalence of AGE in Cuba and other developed countries (8-11). The yearly incidence rate was 1.1 episodes per person-year. When extrapolated to the total population, it corresponded to 79,157.1 episodes of AGE each year, and this is higher than the rates found in several developed countries, For example, the incidence rate for AGE was 0.60 episodes per person-year in Ireland for the period 2000-2001 (10), 0.72 episodes per person-year in the United States for the period 1998-1999 (11), and 0.92 episodes per person-year in Australia for the period 2001-2002 (12).

**Table 11.** Unit cost of services and medical supplies in private and public health systems

Cost variable	Source	Unit cost in public system (Dollar)	Unit cost in private system (Dollar)
Medical visit			
Public	Hospital accounts department/DMA	5*	125 (range 100-150)
Medication			
Pain killer	Central Medical Store	0.05/500 mgx2×3=0.30**	2.00
ORS	Central Medical Store	0.75/packx3=2.25	4.5×3=13.5
Antibiotics	Central Medical Store	0.03/250 mgx3×3×5=1.35***	8.00
Gravol	Central Medical Store	0.05/50 mgx4×2=0.4****	2.00
Loperamide	Private dispensary	0.50/2 mgx2=1.0*****	2.00
IV fluid (0.9% sodium solution)	Central Medical Store	3.42/litre	
Testing of stool specimens	Billing department, PMH	20.00	
Loss of working days			
Caregiver service	Labour standard order (Minimum wage)	28.75/day	
Duration of illness	Population survey	2 days (Median value)	

*It is a flat rate for every visit by the patient at the Princess Margaret Hospital; otherwise it is free in all primary-care facilities. It is not possible to calculate actual cost of medical visit to the public healthcare system;

**Cost of acetaminophen and 1,000 mg 3 times a day/as needed;

***750 mg of Flagyl, 3 times a day for 5 days;

****50 mg dimenhydrinate, 4 times a day for duration of illness (=2 days, median);

*****Loperamide 4 mg initial dose and 2 mg after every loose stool

Although the seasonal difference in prevalence (9.2% in low and 8.0% in high season) was contrary to the data available at the surveillance unit, there was very little variance in AGE surveillance data throughout the study period. Furthermore, public announcements for proper handwashing and hygiene practice during pandemic H1N1 outbreak in Dominica may have impacted these results.

In this study, females were 1.6 times more likely to experience acute diarrhoeal disease than males. This is consistent with other studies (10,13-16) where higher rates were observed in females than males. The reasons for this may be due to differences in route of exposure, such as food preparation (17), or differences in biology. Nonetheless, in-depth studies of the specific reasons for such an increase are needed to implement appropriate prevention measures aimed at decreasing burden of disease in this sub-population.

The highest monthly prevalence of AGE was among the 1-4 year(s) age-group (25.0%), followed by 15-24 years (10.6%) and 5-14 years age-group (10.4%), which is consistent with international findings (9,11,13). Younger children are at a higher risk for AGE due to poor hygienic practices, resulting in ingestion of contaminated food and water (18,19). Overall, the rate of handwashing before meals and after using the toilet, particularly with soap, was not high; this may have contributed to the spread of disease. Although the association between age-group and illness was insignificant (p=0.14), the prevalence of AGE was higher among the under-5 children; similar results of increased risk in children were found in many other studies (10,12). The lack of association may be due to sample-size rather than true lack of association in the population.

**Table 12.** Analysis of basic cost as a consequence of AGE

Cost variable	Multiplier factors from the survey											Annual no. of AGE= 8.6%*12*71,961	Unit cost (EC$) at the public health service	Unit cost (EC$) at the private service	Total cost (EC$) in private system	Total cost (EC$) in public system
	% seeking med. care in private facility	% seeking medical care in public hospitals	% taking pain killer	% taking ORS	% taking antibiotics	% taking antiemetic	% taking antidiarrhoeal drugs	% hospitalized, hence given IV	submitting stool samples	% adult stay home	% needing caregiver service
Medical visit	2.40%	7.10%											5	80	142,585	26,363
																
Medication																
																
Pain killer			4.80%										0.3	1	86	1,069
																
ORS				4.80%									2.25	13.5	1,155	8,020
																
Antibiotic																
					3.60%								1.35	8	513	3,609
(Flagyl)																
Antiemetic																
						1.20%							0.4	2	43	356
(Gravol)																
Antidiarrhoeal																
drug (Loper-							1.10%						1	2	39	817
amide)												74,263				
IV Fluid								0.00%					3.42		0	0
																
Stool testing									2.40%				20			35,646
																
																
Subtotal															1 44,421	75,882
																
Loss of work																
										62.70%			60		0	2,793,774
Median																
1.5*40=60																
Caregiver																
											16.70%		57.5		0	713,110
2*28.75=57.5																
																
																
Subtotal															144,421	3,582,766
																
Total														3,727,187.19 (EC$)	

Prevalence varied according to health districts, with the highest monthly prevalence of self-reported cases of AGE found in Portsmouth Health District and the lowest reported monthly prevalence in Grand Bay Health District. Portsmouth Health District is the second-largest health district situated in the north of the island with a population of 7,956 (1). It is also the largest health district that is populated by diverse ethnic groups, i.e. Haitian, Dominican, East Indian, Chinese, and North American. There are localities within these health districts, characterized by a large number of small and makeshift food outlets. Many of these outlets are set up along the main streets and are operated under poor sanitary conditions. Thus, it may have contributed to a higher prevalence of AGE. Compared to other perceived factors, a high proportion of the respondents (29.8%) with diarrhoeal illness attributed their AGE to food consumption, and 22.6% of cases attributed to drinking-water when asked about the perceived cause of AGE.

Only 16.7% of cases sought medical care despite free healthcare services and supplies within the primary healthcare system, which is significantly lower compared to the study conducted in Cuba (9). The reason for not seeking care may be that the duration of illness was not long enough (median 2 days) to warrant a visit, and/or the illness was not severe enough, which seems to be suggested by the fact that there were no hospitalizations among the respondents. Only 35.7% of those who sought medical care were asked to submit stool samples, and 40% submitted samples. A substantial proportion of cases (44%) who did not seek healthcare treated the symptoms with homemade (bush/herbal) remedies. This indicates that there is still a substantial burden and additional cost associated with cases who do not seek formal medical care.

The predominant pathogen isolated over the study period was norovirus, followed by *Giardia, Salmonella,* and *Shigella.* This was markedly different from the laboratory-based AGE data reported to the national surveillance system, which showed that *Salmonella* was the most common pathogen. This finding has significant implications for our surveillance and intervention measures. Unlike *Salmonella,* norovirus is a highly-contagious viral pathogen. Transmission can occur through many routes, including faecal-oral, person-to-person, by aerosolized faecal material or vomitus and from touching contaminated surfaces. Infected persons continue to shed the pathogen after the symptoms have subsided, and shedding can still be detected many weeks after infection (20). To prevent and control norovirus outbreaks, pathogen-specific prevention guidelines need to be adopted in Dominica.

### Limitations

Several limitations affected this study. Selection bias was evident since the age and gender distributions of the study participants differed from those of the census population (2009 mid-year estimate). Similar to other studies, another potential limitation of this study is recall bias due to reliance on the respondents’ recollection of events over the previous four weeks.

### Conclusions

For the first time in Dominica, this study provides evidence of a significant health burden and distribution of diarrhoeal disease. Overall, 1.1 episodes occur per person-year, with higher rates in females and those aged <5 years in Dominica. These high-risk groups should be considered when allocating resources. The estimated burden of AGE and AGE pathogens is substantially higher than that reported to the Ministry of Health, highlighting the fact that these enteric pathogens still pose a significant health burden. It is essential to design appropriate interventions and to minimize the impact of these pathogens in the population. The findings of this study will be applied to advocate for improved reporting of foodborne pathogens to the Ministry of Health.

## ACKNOWLEDGEMENTS

We thank the Caribbean Epidemiology Centre (CAREC), through its regional BOI coordinator for providing overall technical coordination in this work. We also thank the following institutions and persons for providing technical and financial support: Ross University, the Pan America Health Organization (in particular Dr. Enrique Perez), Public Health Agency of Canada (in particular Dr. Kate Thomas), Global Health Research Initiative (GHRI), and the International Development Research Center (IDRC). The authors also gratefully acknowledge the Ministry of Health, Dominica, for facilitating this study process, and technologists in Princess Margaret Hospital Laboratory (in particular Mr. Eric Carbon) for their contribution and thank residents of Dominica for participation in the study.
